# First trimester serum placental growth factor and hyperglycosylated human chorionic gonadotropin are associated with pre-eclampsia: a case control study

**DOI:** 10.1186/s12884-016-1169-4

**Published:** 2016-11-25

**Authors:** Elina Keikkala, Sini Koskinen, Piia Vuorela, Hannele Laivuori, Jarkko Romppanen, Seppo Heinonen, Ulf-Håkan Stenman

**Affiliations:** 1 Obstetrics and Gynecology, University of Oulu and Oulu University Hospital, Northern Ostrobothnia Hospital District, PB 23, 90029 Oulu, Finland; 2 Obstetrics and Gynecology, University of Helsinki and Helsinki University Hospital, Biomedicum Helsinki, PB 700, 00029 Helsinki, Finland; 3 Obstetrics and Gynecology, Porvoo Hospital, PB 500, 06151 Porvoo, Finland; 4 Medical and Clinical Genetics, University of Helsinki and Helsinki University Hospital, PB 63, 00014 Helsinki, Finland; 5 Institute for Molecular Medicine Finland, University of Helsinki, PB 20, 00014 Helsinki, Finland; 6 Eastern Finland Laboratory Centre, PB 1700, 70211 Kuopio, Finland; 7 Clinical Chemistry, University of Helsinki and Helsinki University Hospital, PB 700, 00029 Helsinki, Finland

**Keywords:** Pre-eclampsia, Small-for-gestational-age, Gestational hypertension, Placental growth factor, Hyperglycosylated human chorionic gonadotropin, Pregnancy-associated plasma protein-A

## Abstract

**Background:**

To study whether maternal serum hyperglycosylated human chorionic gonadotropin (hCG-h) improves first trimester prediction of pre-eclampsia when combined with placental growth factor (PlGF), pregnancy-associated plasma protein-A (PAPP-A) and maternal risk factors.

**Methods:**

Gestational-age-adjusted concentrations of hCG, hCG-h, PlGF and PAPP-A were analysed in serum samples by time-resolved immunofluorometric assays at 8–13 weeks of gestation. The case–control study included 98 women who developed pre-eclampsia, 25 who developed gestational hypertension, 41 normotensive women with small-for-gestational-age (SGA) infants and 177 controls.

**Results:**

Of 98 women with pre-eclampsia, 24 women developed preterm pre-eclampsia (diagnosis < 37 weeks of gestation) and 13 of them had early-onset pre-eclampsia (diagnosis < 34 weeks of gestation). They had lower concentrations of PlGF, PAPP-A and proportion of hCG-h to hCG (%hCG-h) than controls. In receiver-operating characteristics (ROC) curve analysis, the area under the curve (AUC) for the combination of PlGF, PAPP-A, %hCG-h, nulliparity and mean arterial blood pressure was 0.805 (95% confidence interval, CI, 0.699–0.912) for preterm pre-eclampsia and 0.870 (95% CI 0.750–0.988) for early-onset pre-eclampsia. Without %hCG-h the AUC values were 0.756 (95% CI 0.651–0.861) and 0.810 (95% CI 0.682–0.938) respectively. For prediction of gestational hypertension, the AUC for %hCG-h was 0.708 (95% CI 0.608–0.808), but for other markers the AUC values were not significant. None of the AUC values were significant for the prediction of SGA infants in normotensive women.

**Conclusions:**

First trimester maternal serum %hCG-h tended to improve prediction of preterm and early-onset pre-eclampsia when combined with PlGF, PAPP-A and maternal risk factors.

## Background

Pre-eclampsia occurs in 2–8% of all pregnancies. It is a significant cause of maternal morbidity and mortality, especially in developing countries. Pre-eclampsia also increases the risk of pre- and perinatal complications such as intra-uterine growth restriction (IUGR), low birth weight, preterm birth and perinatal death [1].

So far there has been no therapy for pre-eclampsia other than delivery [1]. However, several recent studies suggest that early-onset (diagnosis before 34 weeks of gestation) and severe forms of pre-eclampsia might be prevented by daily low-dose aspirin when started by the 16^th^ week of gestation in mothers at risk [2, 3]. Therefore, early prediction of pre-eclampsia has become important.

As the development of the placenta and its vasculature are often impaired in pre-eclampsia, maternal serum concentrations of placenta-derived factors may be useful in identifying women at risk. Maternal serum concentrations of placental growth factor (PlGF), a member of the vascular endothelial growth factor (VEGF) –family, have been shown to be lower in the first and second trimesters in women who will develop pre-eclampsia as compared to controls [4–8]. Maternal serum concentrations of soluble fms-like tyrosine kinase-1 (sFlt-1), an antagonist of PlGF, are elevated in the second trimester in women with subsequent pre-eclampsia and seem to predict short-term complications of pre-eclampsia in established disease [5, 9, 10]. Pregnancy-associated plasma protein-A (PAPP-A) is used clinically for first trimester screening for Down’s syndrome [11], and low concentrations in maternal serum have also been associated with early-onset pre-eclampsia [4, 12].

The placenta produces various forms of human chorionic gonadotropin (hCG) including hyperglycosylated hCG (hCG-h), which is the major form of hCG in early pregnancy [13]. Elevated concentrations of hCG-h have been observed in gestational trophoblastic diseases [14] and suggested to be associated with increased cytotrophoblast activity [15–17]. We have recently shown that the proportion of hCG-h to total hCG (%hCG-h) at 8–13 weeks of gestation predicts pre-eclampsia with moderate accuracy, i.e. with 56% sensitivity at 90% specificity [12]. Low concentrations of hCG-h in mid-trimester maternal urine, but not in serum have also been associated with subsequent pre-eclampsia [18, 19].

Clinical risk factors include nulliparity, a history of pre-eclampsia, multiple gestation, obesity and chronic hypertension [20–22]. Furthermore, reduced uterine artery blood flow, high first trimester uterine artery pulsatility index (PI) and resistance index (RI), detected by Doppler ultrasound, indicate increased risk of pre-eclampsia [4, 23].

Earlier studies have combined marker concentrations and clinical risk factors in an attempt to predict subsequent pre-eclampsia already in the first trimester. Thus far the most promising combinations in early pregnancy have been those including maternal characteristics, maternal blood pressure and PlGF and/or PAPP-A, and in many studies also uterine artery Doppler PI. Furthermore, these algorithms give the best prediction rates for early-onset pre-eclampsia [4, 6]. Some studies have found that inclusion of sFlt-1 concentrations improves the prediction rates in the cases of late-onset pre-eclampsia [8, 24], but others have found sFlt-1 to not predict pre-eclampsia during the first trimester [7, 9, 25].

The aim of this study was to investigate whether a combination of first-trimester serum %hCG-h with PlGF, PAPP-A and maternal clinical risk factors improves the diagnostic accuracy for prediction of pre-eclampsia.

## Methods

### Patients

Altogether 12,615 pregnant women gave blood samples while attending first trimester screening for Down’s syndrome at 8–13 weeks of gestation in the Kuopio University Hospital region in Finland between April 1^st^, 2008 and December 31^st^, 2010. Gestational age was determined by measuring the crown-rump length of the fetus by ultrasound. Approval to carry out the study was given by the Ethical Research Committee of Kuopio University Hospital. Written informed consent was obtained from all participants.

Criteria of the American College of Obstetricians and Gynecologists (ACOG) were used to define pre-eclampsia: systolic blood pressure ≥ 140 mmHg or diastolic blood pressure ≥ 90 mmHg in two separate measurements at least 6 h apart after 20 weeks of gestation in a previously normotensive woman with a 24-h urinary protein excretion of ≥ 0.3 g [26]. Superimposed pre-eclampsia was defined as onset of proteinuria ≥ 0.3 g/ 24 h in women with chronic hypertension or hypertension before 20 weeks of gestation [26]. Exclusion criteria were multiple gestation or major congenital anomalies.

According to hospital records, 273 women were diagnosed with pre-eclampsia. After exclusion of patients with missing samples (*n* = 109), multiple gestation (*n* = 10) or unverified diagnosis according to definition by ACOG (*n* = 56), 98 women with pre-eclampsia were included in the study. According to the onset of symptoms, two subgroups were analysed separately: early-onset pre-eclampsia (diagnosis < 34 weeks of gestation) and preterm pre-eclampsia (diagnosis < 37 weeks of gestation). Pre-eclamptic women giving birth to small-for-gestational-age (SGA) infants (age- and sex-adjusted birth weight below the 10^th^ percentile [27]) were also analysed separately. In addition, the study included normotensive women with SGA infants and women with gestational hypertension (elevated blood pressure as defined above without proteinuria). We selected controls among women who according to hospital records did not develop pre-eclampsia, gestational hypertension or did not give birth to SGA infants as described in our previous study [12]. Of the 427 controls in the previous study, 177 serum samples were available for analysis for this study.

### Laboratory techniques

Blood was allowed to clot for 30 min at room temperature before separation of serum by centrifugation. Serum samples were stored at +4 °C and analysed for PAPP-A within five days according to manufacturer’s instructions (Perkin Elmer Wallac Oy, Turku, Finland). The samples were then stored at −20 °C until analysis of hCG (Perkin Elmer Wallac Oy, Turku, Finland) and hCG-h by time-resolved fluoroimmunoassay as described previously [12]. Concentrations of PlGF were determined in February 2012 according to manufacturer’s instructions using the AutoDELFIA assay (Perkin Elmer Wallac Oy, Turku, Finland). Intra- and inter-assay coefficients of variation (CV) were <1.8% and <3.7% (PAPP-A), 1.8% (mean) and < 8.8% (hCG), 2.2% and <10.8% (hCG-h) and 4.8% and <8.8% (PlGF). Variation was determined in ten aliquots of two serum pools analysed either in the same or in consecutive runs. The calibrators for PAPP-A covered the range 10–2000 mU/L, for hCG 5–30600 pmol/l, for hCG-h 9–9000 pmol/l and 6–4920 pg/mL for PlGF. Serum samples were diluted 5-fold prior to assay of PAPP-A, 100-fold prior to assays of hCG and hCG-h. This eliminates interference by complement in the hCG-h assay [28]. The PlGF assay measures free PlGF but not PlGF bound to sFlt-1 [29].

### Statistical analysis

The concentrations of PlGF were converted to multiples of median (MoM) values using principal component regression analysis based on the equation “median PlGF log = 0,153* gestational weeks - 0,1157”. Concentrations of hCG-h, PAPP-A and %hCG-h were adjusted for gestational age by converting the concentrations to MoM values as described previously [12]. After log-transformation, the MoM values were normally distributed according to the Kolmogorov-Smirnov test.

Differences between cases and controls were analysed by ANOVA and post hoc comparisons for controls by Dunnett’s test. For continuous variables the Mann–Whitney U test was used to compare clinical characteristics of the groups. Comparison of dichotomized variables was done with the x^2^ test. Multivariate linear regression analysis was used to study correlations between clinical characteristics and biomarker concentrations. Logistic regression was used to analyse the contributions of different maternal risk factors and serum markers to the risk of pre-eclampsia. Receiver-operating characteristic (ROC) curve analysis was used to estimate the diagnostic accuracy, which was expressed as the area under the curve (AUC). SPSS version 21 was used to carry out statistical analysis. The results were considered statistically significant when *P* values were < 0.05. Results were expressed as medians and 95% confidence intervals (CIs) or interquartile range (IQR) or mean ± SD, whichever was most appropriate.

### Study power

The power of the study was calculated according to our previous data. Power analysis was based on the difference of gestational-age-adjusted PlGF concentrations in women with subsequent pre-eclampsia and controls at 14–17 weeks of gestation [19]. For 98 cases of pre-eclampsia and 177 controls the power was 100%, for 13 cases of early-onset pre-eclampsia it was 82% and for 24 cases of preterm pre-eclampsia it was 97% with a two-tailed *P* value of 0.05.

## Results

Of the 98 pre-eclamptic women included in this study, 24 had preterm pre-eclampsia including the 13 with early-onset pre-eclampsia. Twenty of the pre-eclamptic women gave birth to SGA infants (Table 1). Of the patients with early-onset disease 10/13 (77%) gave birth before 34 weeks of gestation. Delivery before 37 weeks occurred in 19/24 (79%) of the women with preterm pre-eclampsia. In addition, the study included 41 normotensive women with SGA infants and 25 women with gestational hypertension.

**Table 1:** Clinical characteristics of the women enrolled

Characteristic	Controls *n* = 177	PE * n* = 98^a^	Early-onset PE *n* = 13^a, a1^	Preterm PE *n* = 24^a^	SGA and PE *n* = 20^a^	GH *n* = 25	SGA *n* = 41
Maternal age (years)	29 ± 5	28 ± 6	29 ± 4	29 ± 5	30 ± 6	32 ± 5^c^	29 ± 5
Nullipara - n (%)	78 (44)	78 (80)^b^	10 (77)^c^	17 (71)^c^	19 (95)^b^	17 (68)^c^	29 (71)^c^
Smokers - n (%)	16 (9)	8 (8)	2 (15)	3 (13)	1 (5)	1 (4)	7 (17)
GA at sampling (weeks)	10.2 ± 0.9	10.4 ± 0.9	10.6 ± 0.8	10.5 ± 0.8	10.4 ± 1	10.1 ± 0.7	10.4 ± 0.8
BMI (kg/m^2^)	25 ± 5	26 ± 5	27 ± 6	26 ± 6	25 ± 5	27 ± 6	23 ± 5
Blood pressure (mmHg)							
Syst. 1st trimester	120 ± 12	125 ± 15^c^	133 ± 22^c^	130 ± 20^c^	122 ± 12	127 ± 13^c^	123 ± 12^c^
Diast. 1st trimester	74 ± 10	78 ± 11^c^	80 ± 14	79 ± 15	76 ± 11	78 ± 11	76 ± 8
Proteinuria (g/24 h)	ND	2.5 (0.7–3.2)	4.8 (1.5–8.5)	4.1 (1.4–5.1)	2.7 (1.3–5.1)	ND	ND
HELLP - n (%)	0	7 (7)^b^	1 (8)^b^	3 (13)^b^	1 (5)^c^	0	0
Chronic disease - n (%)	22(12)	21 (21)^c^	5 (38)^c^	7 (29)^c^	5 (25)	4 (16)	8 (20)
Hypertension - n (%)	0	7 (7)^b^	1 (8)^b^	2 (8)^b^	2 (10)^b^	0	0
Diabetes - n (%)	1(0.6)	4 (4)^c^	3 (23)^b^	4 (16)^b^	0	0	1 (2)
Gestational DM - n (%)	28 (16)	21 (21)	2 (15)	3 (13)	1 (5)	6 (24)	8 (20)
Diagnosis <34 wk -n (%)	0 (0)	13 (13)	13 (100)	13 (54)	2 (10)	2 (8)	0 (0)
Delivery - weeks	40.1 ± 1.1	38 ± 2.9^b^	32.4 ± 3^b^	34.3 ± 3.1^b^	40 ± 2^b^	39.4 ± 1.7^c^	40 ± 0.9
Birth weight (kg)	3.6 ± 0.4	3.0 ± 0.8^b^	1.6 ± 0.6^b^	2.1 ± 0.8^b^	2.3 ± 0.5^b^	3.2 ± 0.6^b^	2.9 ± 0.2^b^
SGA - n (%)	0	20 (20)^b^	2 (15)^b^	6 (25)^b^	20 (100)^b^	8 (32)^b^	41 (100)^b^
Umbilical artery pH	7.24 ± 0.09	7.22 ± 0.09	7.22 ± 0.1	7.22 ± 0.09	7.21 ± 0.1	7.22 ± 0.09	7.23 ± 0.08
Placental weight (g)	620 ± 120	530 ± 150^b^	370 ± 100^b^	430 ± 160^b^	410 ± 110^b^	500 ± 130^b^	540 ± 100^b^

The reported numbers are mean ± standard deviation, amount (percent) or median (interquartile range)

*Abbreviations*: *PE* pre-eclampsia, *early-onset PE* diagnosis made < 34 weeks of gestation, *preterm PE* diagnosis made <37 weeks of gestation (women with early-onset pre-eclampsia included as a subgroup), *GH* gestational hypertension, *SGA* small-for-gestational-age (sex- and age –adjusted birth weight < 10^th^percentile), *GA* gestational age, *ND* not determined, *HELLP* hemolysis, elevated liver enzymes and low platelet count

^a^Some patients may belong to more than one of these subgroups. ^a1^Women with early-onset pre-eclampsia are included in the subgroup of preterm pre-eclampsia. ^b^ *P* < 0.001 compared with controls, or ^c^ *P* < 0.05 compared with controls analysed by Mann Whitney U test for continuous variables or x^2^ test for categorical variables

### Clinical characteristics

Women in the various groups were comparable regarding first-trimester body mass index (BMI), smoking status, and gestational age at sampling. Women with subsequent gestational hypertension were slightly older than the controls. Nulliparity was more common in all affected groups than in controls. Chronic hypertension and type 1 diabetes were more common in pre-eclamptic women than in controls. First trimester systolic blood pressure was higher in women with either pre-eclampsia or SGA without hypertension. Clinical characteristics of the women enrolled are shown in Table 1.

### PlGF, hCG- h and PAPP-A concentrations

The median MoMs of PlGF concentration were lower in women with subsequent preterm pre-eclampsia or pre-eclampsia with SGA infants, as compared to controls. The median MoMs of %hCG-h were lower in all the affected groups, except for normotensive women with SGA infants as compared to controls [12]. Median MoMs of PAPP-A were lower in women with subsequent pre-eclampsia, early-onset pre-eclampsia and preterm pre-eclampsia in comparison to the control group [12] (Table 2).

**Table 2:** Serum marker concentrations

	Controls * n* = 177	PE * n* = 98	Early-onset (<34 wk) PE * n* = 13^a, a1^	Preterm (<37 wk) PE * n* = 24^a^	SGA and PE * n* = 20^a^	GH * n* = 25	SGA * n* = 41
PlGF							
Median	28.4	26.2	25.8	25.3	23.0	26.4	31.3
95% CI	26.7–30.0	24.6–27.3	20.3–28.6	20.3–26.7	20.5–28.4	21.5–27.8	26.9–34.0
Median MoM	1.00	0.98	0.94	0.95^b^	0.97^b^	0.98	1.01
95% CI	0.97–1.02	0.95–1.00	0.88–1.00	0.89–1.00	0.88–0.99	0.92–1.04	0.98–1.05
%hCG-h							
Median	12.4	10.2^c^	8.1^c^	9.1^c^	10.6^c^	10.6^b^	11.6
95% CI	11.7–13.0	8.9–11.1	4.5–9.4	7.3–10.8	7.0–11.6	8.6–11.4	9.7–12.7
Median MoM	1.01	0.92^c^	0.84^c^	0.86^c^	0.85^b^	0.90^b^	0.97
95% CI	0.96–1.02	0.88–0.97	0.70–0.94	0.79–0.97	0.81–0.97	0.82–0.93	0.89–1.06
PAPP-A							
Median	648	567	503	507	499	330^b^	642
95% CI	582–757	463–657	209–769	354–702	337–904	250–690	494–794
Median MoM	1.01	0.97^b^	0.93^b^	0.93^b^	0.96	0.95	0.98
95% CI	0.99–1.03	0.95–1.00	0.81–0.95	0.86–0.97	0.90–0.98	0.91–1.00	0.93–1.02

*PE* pre-eclampsia, *early-onset PE* diagnosis made < 34 weeks of gestation, *preterm PE* diagnosis made < 37 weeks of gestation, *SGA* small-for-gestational-age (sex- and age –adjusted birth weight < 10^th^ percentile), *GH* gestational hypertension, *CI* confidence interval

^a^Some patients may belong to more than one of these subgroups. ^a1^ Women with early-onset pre-eclampsia are included in the subgroup of preterm pre-eclampsia. ^b^ *P* < 0.05 or ^c^ *P* < 0.001 as compared to controls analysed by ANOVA with post hoc Dunnett's test after logarithmic transformation

### AUC values for prediction of pre-eclampsia

**Fig. 1:**
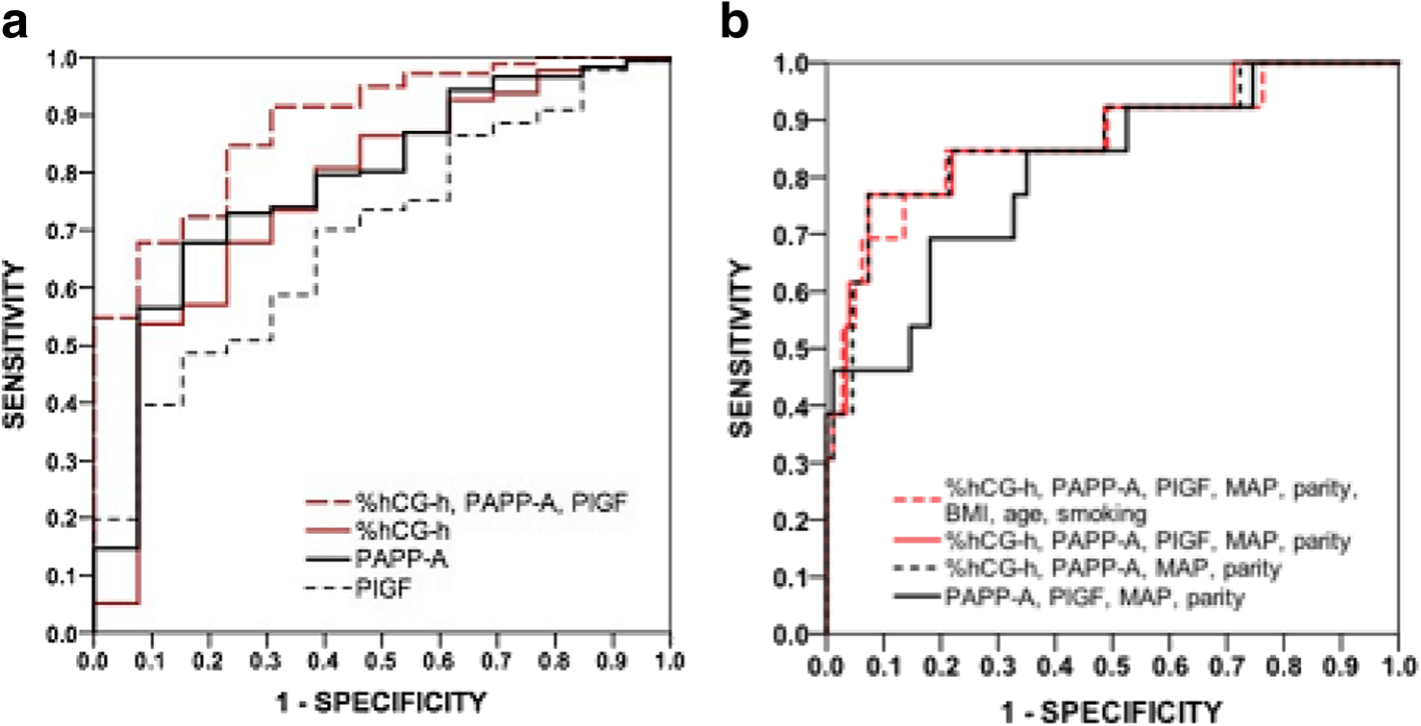
Receiver operating characteristics curves for early-onset pre-eclampsia Receiver operating characteristics (ROC) curves for the MoMs of serum markers (**a**) and their combinations with maternal clinical risk factors (**b**) for the prediction of early-onset pre-eclampsia (diagnosed <34 weeks of gestation)

**Fig. 2:**
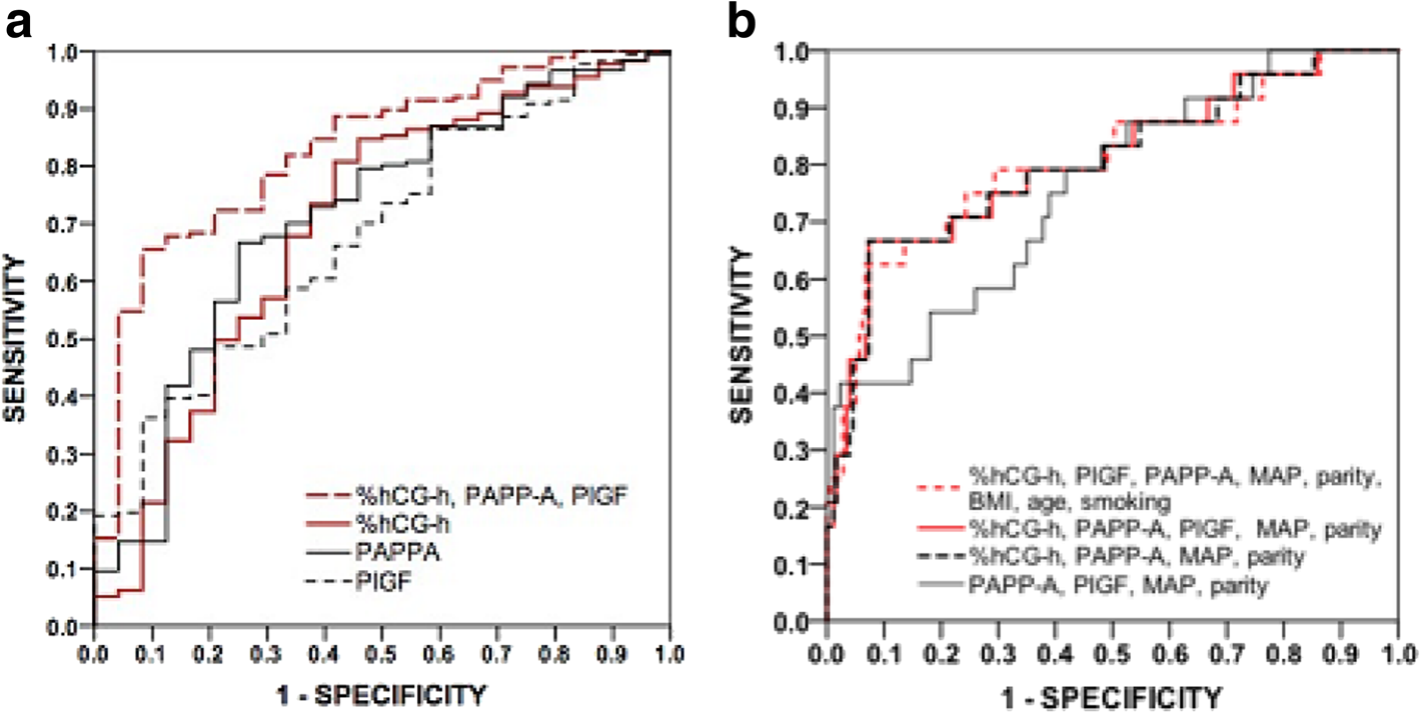
Receiver operating characteristics curves for preterm pre-eclampsia Receiver operating characteristics (ROC) curves for the MoMs of serum markers (**a**) and their combinations with maternal clinical risk factors (**b**) for the prediction of preterm pre-eclampsia (diagnosed <37 weeks of gestation)

**Fig. 3:**
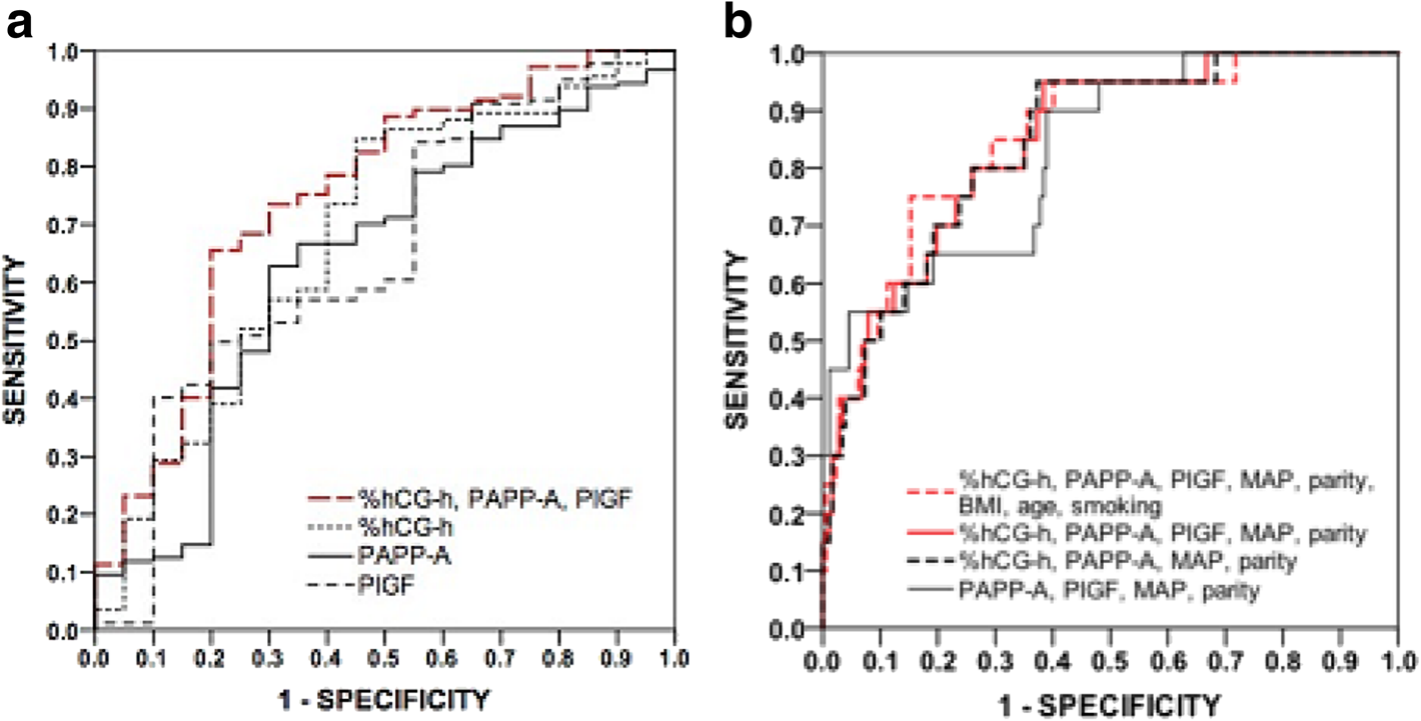
Receiver operating characteristics curves for pre-eclampsia with SGA Receiver operating characteristics (ROC) curves for the MoMs of serum markers (**a**) and their combinations with maternal clinical risk factors (**b**) for the prediction of pre-eclampsia with SGA

The AUC values for prediction of early-onset pre-eclampsia were 0.692 (*P* = 0.021) for PlGF, 0.764 (*P* = 0.001) for %hGC-h, 0.783 (*P* = 0.001) for PAPP-A and 0.885 (*P* < 0.0001) for their combination (Table 3 and Fig. 1a). For prediction of preterm pre-eclampsia the AUC values were 0.680 (*P* = 0.004), 0.699 (*P* = 0.002), 0.714 (*P* = 0.001) and 0.830 (*P* < 0.0001), respectively (Table 3 and Fig. 2a). The corresponding AUC values for pre-eclampsia with SGA infants were 0.653 (*P* = 0.025), 0.682 (*P* = 0.008), 0.634 (*P* = 0.049) and 0.747 (*P* < 0.0001) (Table 3 and Fig. 3a). For prediction of gestational hypertension the AUC value for %hCG-h was 0.708 (95% CI 0.608–0.808, *p* = 0.001), although for other markers the AUC values were not significant. The AUC values were not significant for the prediction of SGA infants in normotensive women (data not shown).

**Table 3:** Area under the curve (AUC) values for MoM values of the markers

	PE * n* = 98	Early-onset (<34 wk) PE *n* = 13^a, a1^	Preterm (<37 wk) PE * n* = 24^a^	SGA and PE *n* = 20^a^
PlGF				
AUC	0.558	0.692^b^	0.680^b^	0.653^b^
95% CI	0.486–0.705	.0.557–0.826	0.572–0.7880	0.519–0.788
%hCG-h				
AUC	0.636^c^	0.764^b^	0.699^b^	0.682^b^
95% CI	0.566–0.705	0.622–0.907	0.577–0.821	0.550–0.815
PAPP-A				
AUC	0.580^b^	0.783^b^	0.714^b^	0.634^b^
95% CI	0.507–0.653	0.656–0.910	0.612–0.849	0.500–0.769
PlGF, %hCG-h, PAPP-A^d^				
AUC	0.689^c^	0.885^c^	0.830^c^	0.747^c^
95% CI	0.622–0.756	0.804–0.966	0.747–0.913	0.624–0.869
PAPP-A, PlGF, MAP, nulliparity^d^				
AUC	0.750^c^	0.810^c^	0.756^c^	0.826^c^
95% CI	0.689–0.811	0.682–0.938	0.651–0.861	0.733–0.919
%hCG-h, PAPP-A, MAP, nulliparity^d^				
AUC	0.776^c^	0.868^c^	0.803^c^	0.843^c^
95% CI	0.717–0.834	0.749–0.986	0.696–0.910	0.760–0.925
%hCG-h,PAPP-A, PlGF, MAP, nulliparity^d^				
AUC	0.776^c^	0.870^c^	0.805^c^	0.845^c^
95% CI	0.718–0.835	0.753–0.988	0.699–0.912	0.763–0.927
%hCG-h, PAPP-A, PlGF, MAP, nulliparity, age, BMI, smoking^d^				
AUC	0.779^c^	0.864^c^	0.806^c^	0.854^c^
95% CI	0.721–0.837	0.740–0.988	0.697–0.914	0.771–0.937

*PE* pre-eclampsia, *early-onset PE* diagnosis made < 34 weeks of gestation, *preterm PE* diagnosis made < 37 weeks of gestation, *SGA* small-for-gestational-age (sex- and age –adjusted birth weight < 10^th^ percentile), *CI* confidence interval, *MAP* mean arterial pressure, *BMI* body mass index

^a^Some patients may belong to more than one of these subgroups. ^a1^Women with early-onset pre-eclampsia are included in the subgroup of preterm pre-eclampsia. ^b^ *P* < 0.05 or ^c^ *P* < 0.001 as compared to controls by ROC analysis. ^d^ Based on a risk calculation derived from logistic regression analysis

The AUC values for combinations of the different markers and maternal risk factors were determined by backward stepwise logistic regression analysis. The risk of pre-eclampsia was calculated with the formula *e* ^L^ / (1 + *e* ^L^), where *e* = 2.718, L = −4.887–1.060 * log PlGF MoM - 9.524 * log PAPP-A MoM - 7.194 * log %hCG-h MoM + 0.034 * mean arterial blood pressure (MAP) + 1.615 * parity (0 = multipara, 1 = nullipara) or *e* ^L^ / (1 + *e* ^L^), where *e* = 2.718, L = −5.617–1.257 * log PlGF MoM – 8.821 * log PAPP-A MoM - 7.126 * log %hCG-h MoM + 0.026 * mean arterial blood pressure (MAP) + 1.736 * parity (0 = multipara, 1 = nullipara) + 0.024 * maternal age + 0.026 * BMI + 0.065 * smoking (0 = no smoking, 1 = smoking).

Combination of %hCG-h, PAPP-A, PlGF, MAP and nulliparity gave an AUC value of 0.870 (*P* < 0.0001) for early-onset pre-eclampsia. When PlGF was left out of the analysis the AUC value was 0.868 (*P* < 0.0001) while the AUC value was 0.810 (*P* < 0.0001) when %hCG-h was removed (Table 3 and Fig. 1b). The respective AUC values for preterm pre-eclampsia were 0.805, 0.803 and 0.756 (*P* < 0.0001) (Table 3 and Fig. 2b). For prediction of pre-eclampsia with SGA infants the respective AUC values were 0.845, 0.843 and 0.826 (*P* < 0.0001) (Table 3 and Fig. 3b). Finally, combining %hCG-h, PAPP-A, PlGF, MAP, nulliparity, age, BMI and smoking gave an AUC value of 0.864 (*P* < 0.0001) for early-onset pre-eclampsia (Table 3 and Fig. 1b), 0.806 (*P* < 0.0001) for preterm pre-eclampsia (Table 3 and Fig. 2b) and 0.854 (*P* < 0.0001) for pre-eclampsia with SGA infants (Table 3 and Fig. 3b).

### Correlations of marker concentrations with clinical characteristics

The concentrations of PlGF (*r* = 0.181) and PAPP-A (*r* = 0.473) increased and those of %hCG-h (*r* = −0.124) decreased with advancing pregnancy. The concentrations of PlGF correlated positively with PAPP-A (*r* = 0.315) and negatively with %hCG-h (*r* = −0.198). The concentrations of PAPP-A correlated negatively with maternal BMI (*r* = −0.191). No statistically significant correlation was observed between BMI and the concentrations of PlGF or %hCG-h. PAPP-A also correlated positively with nulliparity (*r* = 0.173). Smoking correlated with higher concentrations of PlGF (*r* = 0.322). None of the markers correlated with maternal age, chronic diseases, gestational diabetes, first trimester blood pressure, level of proteinuria or placental or infant weight.

## Discussion

As a novel finding we here show that combining %hCG-h with PlGF, PAPP-A and maternal clinical risk factors including nulliparity and first trimester MAP tended to improve prediction of pre-eclampsia as compared to prediction models without %hCG-h. The association of these models, − i.e. lower first-trimester maternal serum concentrations of PlGF and PAPP-A - with subsequent early-onset or preterm pre-eclampsia and pre-eclampsia with SGA infants has also been shown earlier [4–6, 8, 30–32].

Low first trimester serum PlGF concentrations have been suggested to reflect the placental pathology in pre-eclampsia, i.e. in impaired cytotrophoblastic differentiation and invasion and spiral artery formation [33, 34], seen in early-onset or preterm pre-eclampsia; or pre-eclampsia with placental insufficiency or SGA [4, 6, 8, 35]. Also, an imbalance between PlGF and its antagonist sFlt-1 is seen in pre-eclampsia, as administration of sFlt-1 causes pre-eclampsia-like symptoms in pregnant rats [36], and elevated serum concentrations of sFlt-1 are seen from 18 gestational weeks onwards in women with subsequent pre-eclampsia [5, 7, 9]. It is worth noting, though, that changes in maternal serum PlGF and sFlt-1 concentrations are not specific for pre-eclampsia but rather a response to placental stress [37].

We found that smoking correlated with elevated PlGF concentrations, which is in line with earlier observations [38]. Exposure of placental cells in culture to smoke extracts decreases expression of sFlt-1 that may cause elevation of free PlGF in serum [39]. Nicotine stimulates PlGF production in endothelial cells and facilitates endothelial cell migration and tube formation that can be suppressed by sFlt-1 *in vitro* [40]. It may be speculated that these biochemical mechanisms lie behind the negative association between smoking and incidence of pre-eclampsia.

A low %hCG-h seems to be associated with the risk of pre-eclampsia during the first trimester but, as we earlier showed, its ability to predict pre-eclampsia may disappear after the 13^th^ week of gestation [19]. hCG-h is secreted by extravillous cytotrophoblasts and has been suggested to promote cytotrophoblast invasion [15–17]. Thus, a low %hCG-h may reflect the impaired cytotrophoblastic differentiation and invasion in the first trimester seen in pre-eclampsia [33, 41, 42]. This may explain the independent predictive value of %hCG-h in pre-eclampsia, as PlGF reflects the imbalance of the angiogenetic milieu and %hCG-h the failure of cytotrophoblast invasion.

We found that PlGF MoM was lower in women with subsequent preterm pre-eclampsia, pre-eclampsia with SGA infants and in women who developed early-onset disease compared to controls, but statistical significance was not reached in the last group. This may be due to the small sample size as only 13 women developed early-onset pre-eclampsia giving a power of 82% for the study in this setting. We therefore also analysed a group of 24 women that developed preterm pre-eclampsia, which gave a power of 97%.

Gestational hypertension shares clinical risk factors with pre-eclampsia, and one-third of the patients progress to pre-eclampsia [43, 44]. In line with previous studies, we found that PlGF and PAPP-A concentrations did not differ between women who developed gestational hypertension and controls [10, 45]. However, like in pre-eclampsia, %hCG-h was lower in gestational hypertension than in controls. This may indicate that %hCG-h is a more sensitive marker of placental pathology than PlGF or PAPP-A, as similar endothelial dysfunction and impaired cytotrophoblastic invasion that are seen in pre-eclampsia have also been observed in gestational hypertension, only in lesser magnitude [43, 44]. However, pre-eclampsia is a clinically more severe disease as it is associated with more adverse outcomes than gestational hypertension [44].

Normotensive women having SGA infants have been shown to have lower PAPP-A concentrations in some studies [46–49] but, however, in our study we did not observe any differences between cases and controls in PAPP-A or other markers. The explanation might be that SGA infants in our study represented a very mild form of SGA having mean delivering time at term and mean birth weight only slightly below 10^th^ percentile. Concentrations of PlGF in normotensive women with SGA infants have been shown to be lower already in the first trimester as compared to controls [50], but in other studies differences in PlGF concentrations have only been present in the second trimester [51] or not at all [52].

Algorithms combining clinical factors such as nulliparity, maternal age, high BMI and elevated first trimester blood pressure are useful for predicting the risk of pre-eclampsia [6, 20]. When we combined these clinical characteristics with maternal serum concentrations of PlGF, PAPP-A and %hCG-h, the best AUCs were obtained by combining nulliparity and first trimester MAP with the serum markers. As in earlier studies, we observed the highest AUC value (0.870) in early-onset pre-eclampsia. For PlGF alone the AUC value was lower (0.692). In the study of Crovetto et al. the AUC value for first trimester prediction of early-onset pre-eclampsia was 0.788 for PlGF alone and 0.945 for an algorithm combining clinical risk factors, MAP, mean uterine artery PI and PlGF [8]. The role of uterine artery PI is probably of importance in explaining the high AUC value. Goetzinger et al. created a first trimester prediction model with clinical risk factors, uterine artery PI and PAPP-A, and reached an AUC value of 0.76 [53]. The study populations of Crovetto and Goetzinger are similar to ours.

Other studies have found that in first trimester screening algorithms the uterine artery PI has been one of the strongest predictive factors for early-onset pre-eclampsia [6, 8, 54]. Doppler ultrasound measurements were not available in our study population because these are not routinely measured in the first trimester screening appointments in Finland. Uterine artery PI measurements would probably have enhanced the predictive accuracy of our algorithm. On the other hand, Doppler ultrasound is sensitive to inter-observer variation, requires advanced ultrasound equipment and thorough training of the screening personnel. Thus, an algorithm based on marker concentrations and clinical risk factors might be more reproducible and cost-effective than an algorithm including Doppler ultrasound. In present study smoking, maternal age and BMI were less significant in logistic regression analysis, but we also included them in our algorithm since in different study settings they have been shown to have predictive value [6, 35].

A recent systematic review and meta-analysis by Zhong et al. found that the predictive values of first trimester PlGF and PAPP-A were better for early-onset pre-eclampsia as compared to late-onset pre-eclampsia, which is in line with our findings. PlGF was superior to other single markers (PAPP-A, hCG and placental protein 13) but these first trimester markers had low accuracy for prediction of pre-eclampsia. Importantly, the predictive accuracy of first trimester markers was not poorer than that of second trimester markers [55]. In our study we observed that PAPP-A was higher in nulliparous than in multiparous women, which is an unexplained finding also observed in an earlier study [56].

In Finland most pregnant women attend combined first trimester screening for Down’s syndrome, and thus our study population was unselected and represented a wide spectrum of women of different ages and from different backgrounds, which is a strength of our study. There were differences in the clinical characteristics between the subgroups, but this was taken into account in the statistical analysis and found not to affect the results. The small number of women with early-onset pre-eclampsia as well as the fact that uterine artery Doppler measurements were not available are acknowledged weaknesses. Despite these the power of the study was sufficient, and with our algorithm we reached relatively high AUC-values for prediction of early-onset and preterm pre-eclampsia.

In two recent meta-analyses low-dose aspirin treatment has been shown to reduce the risk of early-onset and severe pre-eclampsia in high-risk mothers [2, 3]. ACOG has recently recommended that only a detailed medical history should be used for screening of pre-eclampsia until studies show that aspirin or other interventions reduce the incidence of pre-eclampsia for women at high risk based on first-trimester predictive tests [57]. Therefore, large prospective studies are needed to evaluate whether screening with combinations of PlGF, PAPP-A, %hCG-h and maternal clinical characteristics will be useful for selection of candidates for aspirin treatment.

## Conclusions

First-trimester serum PlGF, PAPP-A and maternal clinical risk factors have been shown to predict early-onset pre-eclampsia. This is the first study to show that combining %hCG-h with PlGF and other above mentioned factors enhance the accuracy to predict early-onset pre-eclampsia.

## Abbreviations

ACOG: American College of Obstetricians and Gynecologists
AUC: Area under the curve
BMI: Body mass index
CI: Confidence intervals
CV: Coefficient of variation
hCG: Human chorionic gonadotropin
hCG-h: Hyperglycosylated hCG
HELLP: Hemolysis, elevated liver enzymes and low platelet count syndrome
IQR: Interquartile range
IUGR: Intrauterine growth restriction
MAP: Mean arterial pressure
MoM: Multiples of median
PAPP-A: Pregnancy-associated plasma protein-A
PI: Pulsatility index
PlGF: Placental growth factor
RI: Resistance index
ROC: Receiver-operating characteristic
sFlt-1: Soluble fms-like tyrosine kinase-1
SGA: Small-for-gestational-age
VEGF: Vascular endothelial growth factor
